# Epidemiological and Clinical Profile of Acute Stroke in Young Adults from a Tertiary Stroke Center in Abu Dhabi—A Retrospective Study

**DOI:** 10.3390/jcm15020727

**Published:** 2026-01-15

**Authors:** Sunitha Bhagavathi Mysore, Sameeha Salim Al Mansoori, Shamma Majed Alhebsi, Noura Ismail Albloushi, Abrar Ali Alshehhi, Jahre Henryson Cuadra Lim, Muhammed Al Jarrah, Cathrine Tadyanemhandu

**Affiliations:** 1 Physiotherapy Department, Fatima College of Health Sciences, Abu Dhabi P.O. Box 3798, United Arab Emirates; sunitha.mysore@actvet.gov.ae (S.B.M.); shammaalhebsi2@gmail.com (S.M.A.); noura.i22@outlook.com (N.I.A.); abrar.ali.a@hotmail.com (A.A.A.); muhammad.aljarrah@actvet.gov.ae (M.A.J.); cathrine.t@actvet.gov.ae (C.T.); 2 Physiotherapy Department, Sheikh Shakhbout Medical City, Abu Dhabi P.O. Box 11001, United Arab Emirates; jahrelim@gmail.com

**Keywords:** stroke, young adults, risk factors, demographics, anthropometrics, clinical profile, function

## Abstract

**Background/Objectives**: Within the last decade, there has been a 19% increase in stroke-related mortality among individuals aged 45–64. Understanding stroke characteristics is crucial, particularly in the younger age groups. This study describes the key demographics and clinical and anthropometric characteristics based on age categories in young adults admitted to the stroke unit in Abu Dhabi. **Methods**: This retrospective observational study had data between October 2024 and March 2025. Data were analyzed descriptively using SPSS, with a more detailed analysis conducted across two age-based groups. **Results**: A total of 51 patients were included, with the median age of 40 (IQR: 37–48) and 44 (86.3%) being males. The median hospital length of stay was 4 days (2–9 days). Most of the patients, 47 (92.2%), had ischemic stroke, with 24 (45.1%) presenting with right-side weakness, and bilateral weakness in 4 (7.8%). The median NIHSS score on admission was 4 (IQR 2–9). Prior to admission, 18 (35.3%) of the patients were known hypertensive, and 12 (23.5%) were diabetic. In terms of anthropometric measurements, the median waist-to-height ratio was 0.58 (0.5–0.69) and BMI was 25.7 (24.2–29.4), with 31 (60.8%) of the patients categorized as either obese or overweight. The statistical significance difference across the age groups was found in the gender distribution only (*p* = 0.034). **Conclusions**: In the UAE, more young men are experiencing Stroke due to lifestyle-related factors, many of which can be prevented. This growing trend calls for early screening, better prevention efforts, and tailored rehabilitation programs.

## 1. Introduction

Stroke affects millions globally, and its rapid increase in prevalence poses significant challenges for healthcare systems in terms of financial, workforce, and healthcare disparities [1]. The burden of stroke continues to grow, with the risk having increased by 50% in the last 20 years [2]. Lifestyle factors play a critical role in determining the age at which individuals experience their first stroke. Recent trends indicate a significant rise in stroke incidence among people aged 30 to 55, largely attributed to unhealthy lifestyle patterns such as a poor diet, physical inactivity, smoking, and chronic stress [3]. The incidence of stroke among young adults has been increasing globally, with heterogeneous rates reported across countries and age groups. For instance, annual increases have been documented at 1.8% in the Netherlands, 5.5% in the United Kingdom, and 1.3% for men and 1.6% for women in Sweden [4,5]. Overall, the rate of increase in stroke incidence among younger populations in the United States of America and European countries is approximately 57% higher compared to older populations [6]. Within the last decade, there has been an increase in stroke-related mortality among individuals aged 45–64, and this is forecasted to be increasing by 2050 [7].

Key risk factors for stroke include high blood pressure, stress, smoking, physical inactivity, excessive alcohol consumption, sleep disturbances, dietary imbalance, and obesity, all of which increase the likelihood of having a stroke before the age of 60 [8,9,10,11,12]. Most younger adults are unaware of their condition and the risk factors associated with stroke [13,14]. Gender differences also play an important role; men tend to have higher rates of smoking and alcohol consumption, while women show an increased prevalence of obesity and hypertension, leading to strokes at older ages [2,15]. In the 45 to 64 age group, women generally have a lower stroke risk compared to men [16,17].

Genetic factors, especially a family history of stroke, significantly contribute to the risk of stroke and are notably prevalent in younger individuals [13]. With an increasing age, anthropometric measurements such as body mass index, waist circumference, and waist-to-hip ratio have been found to be increasingly associated with the stroke risk, as they reflect underlying metabolic and cardiovascular profiles [17,18]. Exposure to ambient particulate matter, a significant environmental pollutant, poses a considerable risk for stroke incidence and mortality [19].

The United Arab Emirates (UAE) is experiencing a high rate of stroke occurrences, and it is the second-leading cause of disability and third-leading cause of death [1]. Risk factors for a stroke are highly prevalent among young adults, yet awareness is low and they are often ignored [20]. This could be due to lifestyle, familial, environmental, and other factors, which need to be identified, as there are limited data published from the UAE [21]. Among the prevalent risk factors for stroke in young adults, individually significant contributors include an elevated systolic blood pressure, overweight or obesity, and type 2 diabetes [7], particularly in low- and middle-income countries, each of which plays a major role in stroke-related morbidity and mortality. Understanding stroke characteristics is important in the Middle East, a region experiencing a rise in stroke incidence, particularly in the younger age groups.

The objective of this study was to describe the key demographics and clinical and anthropometric characteristics based on age categories in young adults admitted to the stroke unit in Abu Dhabi.

## 2. Materials and Methods

This retrospective observational study included patients admitted to the stroke unit of a tertiary care hospital in Abu Dhabi between October 2024 and March 2025. Eligible patients had a confirmed stroke diagnosis and complete demographic, clinical, and lifestyle data. Patients were excluded if they were under 18 years of age, over 55 years, admitted for epilepsy management, or had an unconfirmed diagnosis of stroke.

### 2.1. Setting

This study was conducted within the stroke unit in a large tertiary hospital in Abu Dhabi, comprising 20 beds. The stroke unit is one of the leading state-of-the-art facilities in the country, where patients are admitted for acute care from all over the Emirate. It is one of the very few centers recognized as a center of excellence (CoE) for stroke care. The unit caters to a diverse patient population in terms of age, ethnicity, and severity.

### 2.2. Data Collection

Ethical approval for this study was obtained from the hospital internal review board (Ref: SSMCREC-540). A waiver of informed consent was granted due to the retrospective and observational design of this study. The research team accessed electronic health records during weekday visits to the stroke unit between March and May 2025. Data were extracted from medical records and patient files for all eligible participants using a structured data collection checklist to ensure consistency and completeness.

The checklist was developed based on a review of relevant literature. It covered the following domains: demographics (age, gender, nationality); anthropometric measurements (BMI, waist-to-hip ratio, height, and weight); clinical history (stroke type, length of stay, comorbidities such as hypertension, diabetes, and cardiovascular disease); family history of stroke; and lifestyle factors (sleep patterns, alcohol use, and smoking status). The National Institutes of Health Stroke Scale (NIHSS) was used to quantify the severity of acute stroke at admission, while the Modified Rankin Scale (mRS) was employed to assess functional outcomes at both admission and discharge.

### 2.3. Data Analysis

Data was analyzed using the Statistical Packages for Social Sciences (SPSS) version 30.0.0. Descriptive metrics were reported as the median and interquartile range (IQR) based on normality. Categorical data are presented with frequencies and percentages. For the purposes of the group analysis, patients were categorized into two age groups based on other epidemiological studies: young adults < 45 years and middle-aged adults above 45 years [22]. With the rising incidence of young stroke, its definition varies across studies, with upper age cut-off points ranging from 45 to 59 years [23]. Similarly, the length of stay was categorized based on the days of admission: acute phase: less than 7 days, prolonged stay: ≥7 days.

For BM1, patients were categorized as underweight (BMI < 18.5), normal (BMI 18.5 to <25), overweight (BMI 25 to <30), obesity (BMI 30 to <35) [24].

Waist circumference was categorized as follows: for men, ≤94 cm indicated a low risk, 94–102 cm indicated an increased risk, and ≥102 cm indicated a substantially increased risk. For women, ≤80 cm indicated a low risk, 80–88 cm indicated an increased risk, and ≥88 cm indicated a substantially increased risk. For the waist-to-height ratio, a cut of less than 0.5 was used for no increased risk and more than 0.5 for increased risk [25].

HbA1c categories were defined as follows: normal (<5.7%), pre-diabetes (5.7–6.4%), and high (≥6.5%).

For blood pressure, a reading of 140/90 mmHg or higher was considered elevated. For the mRS, scores of 0–2 were classified as indicating good functional outcomes, whereas scores of 3–5 were considered poor functional outcomes. The NIHSS score was categorized as 0: no stroke symptoms; 1–4: minor stroke; 5–15: moderate stroke; 16–20: moderate to severe stroke; 21–42: severe stroke.

As the continuous data were not normally distributed, non-parametric tests were applied. Associations between categorical data were analyzed using Fisher’s exact test with the statistical significance level set at *p* < 0.05. Cases with missing/unknown values were excluded from the association analysis.

## 3. Results

### 3.1. Demographic Details of the Patients

A total of 51 patients were included in this study, with males numbering 44 (86.3%) and females 7 (13.7%). The age range was 23 to 54 years, with the median age of 40 (IQR: 37–48) with 37 (72.5%) being married, while 12 (23.5%) were single and 2 (3.9%) divorced. Most were Asians (38, 74.5%), living in the UAE with friends or family (36, 70.6%).

The majority of the patients (40, 78.4%) were discharged within the UAE and 11 (21.6%) to their home country. In terms of employment status, 31 (60.8%) were employed in outdoor jobs, 14 (27.5%) had indoor occupations, and 6 (11.8%) did not have a recorded work setting. Of those employed, 28 (54.9%) were in blue-collar jobs.

The sleep pattern of patients was mostly regular 31 (60.8%). Regarding the smoking history, 23 (43.1%) were current smokers, 27 (52.9%) non-smokers, and 1 (2%) a former smoker. Alcohol consumption was low, with only 4 (7.8%) reporting alcohol use.

Based on the age categories in Table 1, gender was the only variable that showed a significant difference between the two groups (*p* = 0.034) (Table 1).

### 3.2. Clinical History and Presentation of the Patients

**Figure 1:**
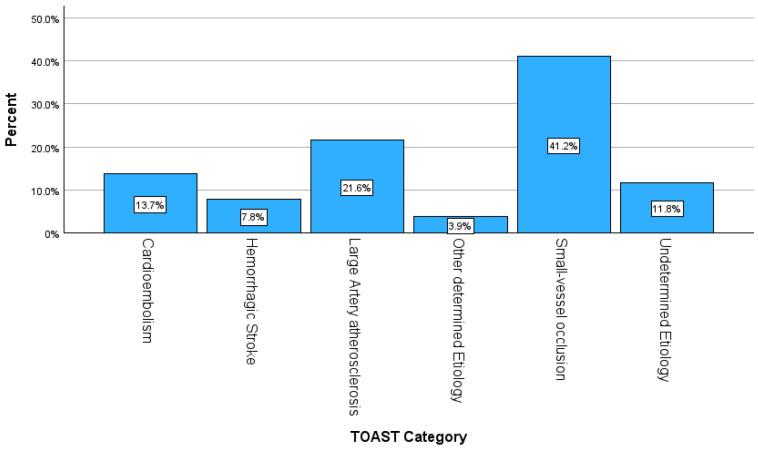
Types of strokes by the specific category.

Only one patient had a previous history of stroke. The majority were classified as ischemic strokes (47, 92.2%), and 4 (7.8%) were a hemorrhagic stroke. The most common type of ischemic stroke was small-vessel occlusion, in 21 (41.2%) of patients, with 11 (21.6%) having large-artery atherosclerosis (Figure 1). Four (7.8%) of the patients had a previous history of transient ischemic attacks (TIAs). All 51 (100%) patients underwent a computed tomography (CT) scan of the brain. In addition, magnetic resonance imaging (MRI) of the brain was performed in 48 (94.1%) patients, and a computed tomography angiogram (CT angiogram) was conducted in 49 (96.1%) patients.

Of the 51 patients assessed for their perfusion status, 31 (60.8%) had no perfusion intervention. Intravenous thrombolysis (IVT) was performed in nine (17.6%) patients, and endovascular therapy (EVT) in eight (15.7%) patients. A combined approach of IVT and EVT was used in three (5.9%) patients.

The median NIHSS score on admission was 4 (IQR 2–9). The most frequent NIHSS category at admission was minor stroke (NIHSS 1–4), observed in 22 (43.1%) patients. This was followed by moderate stroke (NIHSS 5–15) in 19 (37.3%) patients. No stroke symptoms (NIHSS = 0) were recorded in five (9.8%), severe stroke (NIHSS 21–42) occurred in three (5.9%), and moderate-to-severe stroke (NIHSS 16–20) was seen in two (3.9%) patients. The median mRS severity score at admission and at discharge remained the same (3, IQR 1–4).

Right-sided weakness was reported in 24 (45.1%) and bilateral weakness in 4 (7.8%).

In terms of hypertension, known hypertensives prior to admission totaled 18 (35.3%), but on admission, many more presented with high blood pressure (36, 70.6%) when compared to discharge (13, 25.5%).

Similarly, patients who were known diabetics prior to admission totaled 12 (23.5%), but based on the Hb1Ac scores, 15 (29.5%) fell into the category of pre-diabetes and 19 (37.3%) were classified as high. From a total of 51, only 10 (19.6%) of the patients reported being on medication for either hypertension, diabetes, or both prior to stroke.

The median hospital length of stay was 4 days (2–9 days). While not statistically significant, notable differences emerged between groups, including laterality (*p* = 0.069) and blood pressure on discharge (*p* = 0.093) (Table 2).

### 3.3. Anthropometric Measurements of the Patients

In terms of anthropometric measurements, the median waist circumference was 95.3 (89.9–117.5 cm), waist-to-height ratio was 0.58 (0.5–0.69), and BMI was 25.7 (24.2–29.4). Out of 51 patients, the waist circumference was categorized as unknown in 13 (25.5%), increased in 19 (37.3%), and low in 19 (37.3%) patients. For the weight-to-height ratio, 13 (25.5%) had an unknown waist-to-height ratio category, 34 (66.7%) were classified as having an increased waist-to-height ratio, and 4 (7.8%) had no increased risk. Of the 51 patients, 20 (39.2%) had a normal BMI, 22 (43.1%) were overweight, and 9 (17.6%) were classified as obese. There was no significant difference in BMI and waist circumference-based cardiometabolic risk between young adults and middle-aged adults (*p* = 1.000). Both groups demonstrated a similar distribution of overweight/obese and increased-risk waist circumference, respectively (Table 3).

## 4. Discussion

The findings of this six-month retrospective analysis showed that more males were affected with stroke than females, which correlated with other studies in different geographical areas [1,26,27]. The male predominance in this study could mainly be due to demographic and occupational factors, as the UAE has a high number of male expatriates working in blue-collar jobs, which is similar to other countries in the Middle East and North Africa (MENA) region [28,29,30]. This trend is consistent across the Gulf Cooperation Council (GCC), suggesting a regional pattern of male-to-female ratios with two-thirds of the patients in both Saudi Arabia and Qatar [30,31].

Expatriates from the Asian subcontinent form the largest nationality group in our stroke cohort, with similar rates in the other Gulf regions being reported [31,32]. The diversity of nationalities and ethnicities in the UAE may add to the complexity of understanding health behaviors, risk factors, stroke characteristics, and outcomes [29,33]. One of the major challenges has been limited follow-ups, especially with expatriate patients, who would be repatriated to their home countries after discharge from the stroke unit.

Our study found that most patients were in blue-collar jobs compared to white-collar roles. While this difference was not statistically significant, it could be attributed to the small sample size. However, previous studies have shown that occupations with demanding long working hours, extended exposure to the sun, and certain toxins or chemicals are associated with an increased risk of stroke [34,35].

The existing literature suggests that the marital status influences the stroke prevalence and outcome, as married individuals often benefit from greater social support, leading to better results than unmarried individuals [36,37]. Three-quarters of our study participants were married; however, as many were expatriates working in blue-collar jobs, they often lived with friends rather than family. While most patients lived with family or friends when compared to living independently, their pre-stroke living situation did not impact stroke prevalence or severity. This result is consistent with prior studies suggesting that living arrangement alone does not adequately reflect social isolation, a known stroke risk factor [38,39,40].

Approximately half of the patients in our study reported a history of smoking, a well-established risk factor for stroke. Although detailed information on the smoking duration and pack-years was not available, these parameters are critical for accurately assessing the stroke risk [10,41]. Further, the evidence suggests that smoking 20 or more cigarettes per day increases the risk of stroke by approximately fivefold [42]. While alcohol consumption was minimal among our patients, a high alcohol intake is recognized to increase the risk of stroke incidence, but in moderation, it helps in preventing the incidence [43].

Our data showed that about a third of the patients had a known history of hypertension prior to stroke. However, nearly two-thirds of patients presented with high blood pressure on admission, a score that decreased by discharge. The prevalence of hypertension is alarmingly high. Hypertension has been noted to be the single biggest risk factor for stroke, yet the majority of the patients are unaware of their condition [44]. In a large UAE cohort study of 5137 young adults, the prevalence of hypertension among men was three times higher than among women [45]. Across the GCC region, stroke-related deaths and disability-adjusted life years (DALYs) linked to high systolic blood pressure have shown a consistent decline over the past three decades, with a statistically significant reductions observed for both outcomes (deaths: *p* = 0.023; DALYs: *p* = 0.039) [46].

Similarly, diabetes mellitus was prevalent among the patients studied. These figures are notably high for a population under 55 years old and demonstrate the early onset of cardiometabolic disease in this group [45]. Both conditions are known to contribute significantly to stroke pathogenesis, especially when poorly controlled or undiagnosed. This highlights the importance of enhancing community awareness and implementing systematic monitoring, as these represent modifiable risk factors for stroke.

The BMI distribution in our study showed that a large percentage of patients were in the overweight and obese category, particularly the youngest age. The waist-to-height ratio indicated an increased risk of stroke in more than half of our patients. Waist circumference risk and BMI did not differ between the young and middle-aged participants. Both age groups had comparable proportions of individuals with increased abdominal obesity and general obesity, suggesting that these factors are relevant risk factors across age ranges, not only in older patients. The waist circumference is a central factor in the clinical profile of young stroke patients, and it may be a strong predictor of the stroke severity or outcomes, possibly due to its association with metabolic syndrome or cardiovascular risk. This is consistent with other studies that have reported an association between a high risk of stroke and increased BMI, as well as larger waist and neck circumferences [5,47]. A longer duration of residence in the UAE was significantly associated with central obesity. Individuals living in the country for 6–10 years had 63% higher odds of being classified with central obesity, while those residing for more than 10 years had nearly double the odds, compared to those who had lived in the UAE for only 1–5 years [33].

Atherosclerotic disease accounts for approximately one-third of ischemic strokes in young adults aged 15–45 years, with both small- and large-vessel involvement becoming increasingly common from around age 30. This pattern was consistent with our findings, where small-vessel and large-vessel involvement represented the highest proportions of ischemic stroke within this population [48].

The ability to draw inferences about the length of stay was restricted by the small sample size. The length of stay varies based on various factors, including age, rehabilitation needs, and other comorbidities [49]. In our study, the change in mRS score was minimal from admission to discharge, indicating an improvement in some functional status, particularly among young adults. Although not statistically significant, this improvement may reflect timely, high-quality care in a specialized stroke unit [50,51]. The minimal change is likely due to the short stay in the acute care center, which allows stabilization rather than substantial functional recovery. The discharge destinations for our patients included post-acute care facilities, home (with or without homecare services), and outpatient departments. This pattern is consistent with discharge practices reported in other GCC countries [13].

An individualized, tailored approach is significantly more effective than a generic strategy in reducing risk factors for stroke and cardiovascular conditions among blue-collar workers [52]. Preventive strategies should therefore prioritize personalized interventions—such as targeted lifestyle counseling, regular health screenings, and culturally adapted education programs. Primary stroke prevention strategies for the general population include adopting a balanced diet rich in fruits, vegetables, and fish while limiting saturated fats; engaging in regular physical activity; quitting smoking; moderating alcohol consumption; and managing stress effectively [8,52,53]. Furthermore, timely initiation of pharmacotherapy is essential once risk factors are identified. Delaying treatment for conditions such as hypertension, hyperlipidemia, and hyperglycemia significantly increases the risk of stroke [50,54].

One of the key challenges in identifying stroke risk factors among our expatriate population is the limited information available on their lifestyle patterns and their frequent reluctance to monitor the health status regularly. Addressing these gaps would make it easier to implement effective preventive measures [55,56]. The documentation used for data collection did not include a measure of physical activity levels, which is an important parameter for assessing risk factors. In hyperacute settings, it would not have been feasible to collect this data. Future research should consider incorporating this measure during the prehabilitation phase or in post-hyperacute care [53].

Given the limited number of stroke centers of excellence in the UAE, health authorities have called for the expansion of 24/7 stroke centers around the country and the development of tele-stroke networks to enable a rapid diagnosis and timely intervention. Furthermore, pragmatic solutions are essential to address critical gaps in workforce capacity-building and to strengthen robust epidemiological surveillance systems.

There were several limitations of this study. The sample size and one single hospital limited our ability to draw inferences on risk factors and generalizability to the whole population in the UAE. Because this was a retrospective study, several key details were either incomplete or missing, specifically comprehensive data on smoking, physical activity, diet, occupational stress, and the time from stroke onset to hospital admission (hours). This limitation potentially impacts the interpretation of the results. The hospital’s location and the predominance of male patients with blue-collar occupations in the data collection process may have introduced selection bias and gender structure bias within this study.

Future studies should consider the stroke registry and the wider population. More longitudinal studies examining the continuum from prehospital care to rehabilitation outcomes in the region are recommended, with particular attention to the age at onset within the studied populations. Also, the critical information that need to be considered are the time from the onset of initial symptoms of stroke to hospital presentation, and detailed data related to the lifestyle, specifically on smoking, physical activity, diet, and occupational stress. Comprehensive immediate and acute management of these patients, including the rehabilitation outcomes, should further be explored.

## 5. Conclusions

Stroke has traditionally been associated with the elderly, but this research confirms that stroke is becoming increasingly common among younger adults, particularly blue-collar workers in their thirties and forties. Most patients presented with right-sided weakness, and more than half were discharged with poor functional outcomes. Although many patients showed some improvement during their hospital stay, a significant number had disabilities. This highlights the importance of strong rehabilitation programs and consistent follow-up care after discharge to support long-term recovery. A substantial number of patients had high blood pressure and Hb1Ac scores during the hospital stay. A considerable proportion of patients in this study presented with an elevated BMI and increased waist-to-height ratios. The findings justify routine screening for risk factors in younger adults, which include hypertension, diabetes, obesity, and lifestyle factors, as early detection and intervention play a significant role in stroke prevention. There is an urgent need to implement effective stroke prevention strategies, prioritizing population-wide interventions such as national campaigns promoting lifestyle modifications, incentivizing workplaces to adopt wellness programs, and expanding the use of evidence-based mobile and telehealth platforms.

## Figures and Tables

**Table 1:** Demographics details of the patients according to age group (N = 51).

Variable	Attribute, n (%)	Young Adults	Middle-Aged	Test
Gender	Male	32 (72.7)	12 (27.3)	*p* = 0.034 *
	Female	2 (28.6)	5 (71.4)	
Marital status ^a^	Married	23 (62.2)	14 (37.8)	*p* = 0.334
	Not Married	11 (78.6)	3 (21.4)	
Nationality ^a^	Asia	27 (71.1)	11 (28.9)	*p* = 0.315
	MENA and Africa *	7 (53.8)	6 (46.2)	
Living situation before stroke	Family/Friends	24 (66.7)	12 (33.3)	*p* = 0.746
	Independent	8 (61.5)	5 (38.5)	
Discharge destination ^a^	Family/Friends/Home Country *	22 (59.5)	15 (40.5)	*p* = 0.284
	Independent	9 (81.8)	2 (18.2)	
Occupation ^a^	Blue Collar	20 (71.4)	8 (38.7)	*p* = 0.366
	White Collar	11 (85.7)	8 (14.3)	
Sleep pattern ^a^	Regular	19 (61.3)	12 (38.7)	*p* = 0.385
	Irregular	6 (85.7)	1 (14.3)	
History of smoking	Yes	14 (58.3)	10 (41.7)	*p* = 0.254
	No	20 (74.1)	7 (25.9)	
History of alcohol	Yes	4 (100)	0	*p* = 0.288
	No	30 (63.8)	17 (36.2)	

* Statistical significance. ^a^ Cases with missing/unknown values were excluded from the association analysis or recoded as follows—marital status, single and divorced were merged; nationalities were collapsed into two groups: Asia vs. MENA + Africa (including Sierra Leone, Cameroon, Ethiopia); discharge destination was dichotomized into supported (family/friends or home country) vs. independent to ensure adequate cell counts and reflect social support status.

**Table 2:** Clinical history and presentation of the patients according to age groups (N = 51).

Variable	Attribute, n (%)	Young Adults	Middle-Aged	Test
Laterality ^a^	Right	21 (77.8)	6 (22.2)	*p* = 0.069
	Left	11 (50.0)	11 (50.0)	
Length of stay	Acute (<7 days)	22 (66.7)	11 (33.3)	*p* = 1.00
	Prolonged (≥7 days)	12 (66.7)	6 (33.3)	
NIHSS on admission ^a^	Mild	19 (70.4)	8 (29.6)	*p* = 0.569
	Moderate–severe	15 (62.5)	9 (37.5)	
mRS on admission	Good	12 (63.2)	7 (36.8)	*p* = 0.763
	Poor	22 (68.8)	10 (31.2)	
mRS on discharge	Good	17 (70.8)	7 (29.2)	*p* = 0.767
	Poor	17 (63.0)	10 (37.0)	
Known hypertension prior to stroke	Yes	11 (61.1)	7 (38.9)	*p* = 0.551
	No	23 (67.7)	10 (30.3)	
BP on admission	High	22 (61.1)	14 (38.9)	*p* = 0.328
	Normal	12 (80.0)	3 (20.0)	
BP on discharge	High	6 (46.2)	7 (53.8)	*p* = 0.093
	Normal	28 (73.7)	10 (26.3)	
Known diabetes prior to stroke	Yes	7 (58.3)	5 (41.7)	*p* = 0.503
	No	27 (69.2)	12 (30.8)	
Hb1Ac categories ^a^	High	23 (67.6)	11 (32.4)	*p* = 1.00
	Normal	10 (71.4)	4 (28.6)	

^a^ Cases with missing/unknown values were excluded from the association analysis or recoded as follows—laterality was recoded based on the affected cerebral hemisphere and the expected contralateral side of weakness; NIHSS was recoded to mild for 0–5 scores and moderate–severe for above 6 scores; high and pre-diabetes were combined into a single group labeled abnormal, while normal remained as the second category.

**Table 3:** Anthropometrics of the patients according to age groups (N = 51).

Variable	Attribute, n (%)	Young Adults	Middle-Aged	Test
BMI ^a^	Normal	13 (65.0)	7 (35.0)	*p* = 1.00
	Overweight/Obese	21 (67.7)	10 (32.3)	
Waist–height ratio ^a^	No increased risk	3 (75.0)	1 (25.0)	*p =* 1.000
	Increased	22 (64.7)	12 (35.3)	
Waist circumference ^a^	Low	14 (73.7)	5 (26.3)	*p* = 0.495
	Increased risk	11 (57.9)	8 (42.1)	

^a^ Cases with missing/unknown values were excluded from the association analysis or recoded as follows—BMI categories were collapsed into two groups: ‘normal’ vs. ‘overweight/obese’; for waist circumference, risk categories were collapsed into two groups: ‘low’ and ‘elevated risk’—combining increased risk and substantially increased.

## Data Availability

Data and materials supporting the findings of this study are readily available from the corresponding author upon request. The availability of the data is restricted to investigators based at the tertiary hospital.

## Abbreviations

The following abbreviations are used in this manuscript:

SPSS	Statistical Packages for Social Sciences
mRS	Modified Rankin Scale
IQR	Interquartile Range
BMI	Body Mass Index
UAE	United Arab Emirates
TIA	Transient Ischemic Attack
Hb1Ac	Glycated Hemoglobin Test
GCC	Gulf Cooperation Council
MENA	Middle East and North Africa

## References

[1] Feigin V.L., Norrving B., Mensah G.A. (2017). Global Burden of Stroke. Circ. Res..

[2] Aslam A., Khan U., Niazi F., Anwar I. (2022). Etiology and risk factors of stroke in young adults: A multicentric study. Ann. Med. Surg..

[3] Jacob M.A., Ekker M.S., Allach Y. (2022). Global Differences in Risk Factors, Etiology, and Outcome of Ischemic Stroke in Young Adults—A Worldwide Meta-analysis: The GOAL Initiative. Neurology.

[4] Ekker M.S., Verhoeven J.I., Vaartjes I., Van Nieuwenhuizen K.M., Klijn C.J.M., De Leeuw F.-E. (2019). Stroke incidence in young adults according to age, subtype, sex, and time trends. Neurology.

[5] Li L., Scott C.A., Rothwell P.M. (2022). Association of Younger vs Older Ages with Changes in Incidence of Stroke and Other Vascular Events, 2002–2018. JAMA.

[6] Scott C.A., Li L., Rothwell P.M. (2022). Diverging Temporal Trends in Stroke Incidence in Younger vs Older People: A Systematic Review and Meta-analysis. JAMA Neurol..

[7] Feigin V.L., Owolabi M.O., Feigin V.L. (2023). Pragmatic solutions to reduce the global burden of stroke: A World Stroke Organization–Lancet Neurology Commission. Lancet Neurol..

[8] Markidan J., Cole J.W., Cronin C.A., Merino J.G., Phipps M.S., Wozniak M.A., Kittner S.J. (2018). Smoking and Risk of Ischemic Stroke in Young Men. Stroke.

[9] Jayedi A., Ghomashi F., Zargar M.S., Shab-Bidar S. (2019). Dietary sodium, sodium-to-potassium ratio, and risk of stroke: A systematic review and nonlinear dose-response meta-analysis. Clin. Nutr..

[10] Pan B., Jin X., Jun L., Qiu S., Zheng Q., Pan M. (2019). The relationship between smoking and stroke: A meta-analysis. Medicine.

[11] Wang X., Huang Y., Chen Y., Yang T., Su W., Chen X., Yan F., Han L., Ma Y. (2022). The relationship between body mass index and stroke: A systemic review and meta-analysis. J. Neurol..

[12] Reddin C., Murphy R., Hankey G.J., Judge C., Xavier D., Rosengren A., Ferguson J., Alvarez-Iglesias A., Oveisgharan S., Iversen H.K. (2022). Association of Psychosocial Stress with Risk of Acute Stroke. JAMA Netw. Open.

[13] Al-Senani F., Al-Johani M., Salawati M., ElSheikh S., AlQahtani M., Muthana J., AlZahrani S., Shore J., Taylor M., Ravest V.S. (2019). A national economic and clinical model for ischemic stroke care development in Saudi Arabia: A call for change. Int. J. Stroke Off. J. Int. Stroke Soc..

[14] Alhubail F.M., Al-Mousa A.M., Albusaad R., Alsumaeel S., Alabbadi M.S., Almulhim M.A., Alnaaim S. (2024). Knowledge of Symptoms, Risk Factors, and Treatment Centers of Stroke among the General Population of Al-Ahsa, Saudi Arabia. Ann. Afr. Med..

[15] Bailey R.R., Phad A., McGrath R., Haire-Joshu D. (2018). Prevalence of five lifestyle risk factors among U.S. adults with and without stroke. Disabil. Health J..

[16] Lutski M., Zucker I., Shohat T., Tanne D. (2017). Characteristics and Outcomes of Young Patients with First-Ever Ischemic Stroke Compared to Older Patients: The National Acute Stroke ISraeli Registry. Front. Neurol..

[17] Brauer M., Roth G.A., Aravkin A.Y., Zheng P., Abate K.H., Abate Y.H., Abbafati C., Abbasgholizadeh R., Abbasi M.A., Abbasian M. (2024). Global burden and strength of evidence for 88 risk factors in 204 countries and 811 subnational locations, 1990–2021: A systematic analysis for the Global Burden of Disease Study 2021. Lancet.

[18] Ranjan R., Adhikary D., Ken-Dror G., Yusuf M.A., Moureen A., Hakim M., Sharma P. (2024). Anthropometric measurements in predicting haemorrhagic stroke among Bangladeshi population: The MAGPIE study. J. Multidiscip. Healthc..

[19] Toubasi A., Al-Sayegh T.N. (2023). Short-term Exposure to Air Pollution and Ischemic Stroke: A Systematic Review and Meta-analysis. Neurology.

[20] Ekker M.S., Verhoeven J.I., Schellekens M.M.I., Boot E.M., Alebeek M.E., Brouwers P.J.A.M., Arntz R.M., Dijk G.W., Gons R.A.R., Uden I.W.M. (2023). Risk Factors and Causes of Ischemic Stroke in 1322 Young Adults. Stroke.

[21] El-Hajj M., Salameh P., Rachidi S., Hosseini H. (2016). The epidemiology of stroke in the Middle East. Eur. Stroke J..

[22] Cabral N.L., Nagel V., Conforto A.B., Amaral C.H., Venancio V.G., Safanelli J., Ibiapina F., Longo A.L., Zetola V.D.H.F. (2018). Five-year survival, disability, and recurrence after first-ever stroke in a middle-income country: A population-based study in Joinvile, Brazil. Int. J. Stroke.

[23] Abedi V., Lambert C., Chaudhary D., Rieder E., Avula V., Hwang W., Li J., Zand R. (2023). Defining the Age of Young Ischemic Stroke Using Data-Driven Approaches. J. Clin. Med..

[24] Doehner W., Schenkel J., Anker S.D., Springer J., Audebert H.J. (2013). Overweight and obesity are associated with improved survival, functional outcome, and stroke recurrence after acute stroke or transient ischaemic attack: Observations from the TEMPiS trial. Eur. Heart J..

[25] Browning L.M., Hsieh S.D., Ashwell M. (2010). A systematic review of waist-to-height ratio as a screening tool for the prediction of cardiovascular disease and diabetes: 0·5 could be a suitable global boundary value. Nutr. Res. Rev..

[26] Feigin V.L., Stark B.A., Johnson C.O., Roth G.A., Bisignano C., Abady G.G., Abbasifard M., Abbasi-Kangevari M., Abd-Allah F., Abedi V. (2021). Global, regional, and national burden of stroke and its risk factors, 1990–2019: A systematic analysis for the Global Burden of Disease Study 2019. Lancet Neurol..

[27] Vyas M.V., Fang J., Kapral M.K., Yu A.Y.X., Austin P.C. (2022). Gender inequality in source country modifies sex differences in stroke incidence in Canadian immigrants. Sci. Rep..

[28] Abdu H., Seyoum G. (2022). Sex Differences in Stroke Risk Factors, Clinical Profiles, and In-Hospital Outcomes Among Stroke Patients Admitted to the Medical Ward of Dessie Comprehensive Specialized Hospital, Northeast Ethiopia. Degener. Neurol. Neuromuscul. Dis..

[29] Jaberinezhad M., Farhoudi M., Nejadghaderi S.A., Alizadeh M., Sullman M.J.M., Carson-Chahhoud K., Collins G.S., Safiri S. (2022). The burden of stroke and its attributable risk factors in the Middle East and North Africa region, 1990–2019. Sci. Rep..

[30] Khalil Hussien A., Khalid Alshehri A., Khalid Alanazi F., Mohammed Aljabal A., Ibrahim Alanazi A., Mohammed Alqayidi A., Hussein Alghamdi I. (2024). Characterization of Demographic, Clinical, and Laboratory Risk Factors for Stroke in a Tertiary Hospital in Riyadh, Saudi Arabia. Cureus.

[31] Bhutta Z.A., Akhtar N., Pathan S.A., Castrèn M., Harris T., Ganesan G.S., Kamran S., Thomas S.H., Cameron P.A., Azad A. (2024). Epidemiological profile of stroke in Qatar: Insights from a seven-year observational study. J. Clin. Neurosci..

[32] Khan M., Wasay M., O’Donnell M.J., Iqbal R., Langhorne P., Rosengren A., Damasceno A., Oguz A., Lanas F., Pogosova N. (2023). Risk Factors for Stroke in the Young (18–45 Years): A Case-Control Analysis of INTERSTROKE Data from 32 Countries. Neuroepidemiology.

[33] Shah S.M., Loney T., Dhaheri S.A., Vatanparast H., Elbarazi I., Agarwal M., Blair I., Ali R. (2015). Association between acculturation, obesity and cardiovascular risk factors among male South Asian migrants in the United Arab Emirates–a cross-sectional study. BMC Public Health.

[34] Huynh T., McClure L.A., Howard V.J., Stafford M.M., Judd S.E., Burstyn I. (2022). Duration of employment within occupations and incident stroke in a US general population cohort 45 years of age or older (REGARDS study). Am. J. Ind. Med..

[35] Fadel M., Sembajwe G., Jian L., Leclerc A., Pico F., Schnitzler A., Roquelaure Y., Descatha A. (2024). O-346 association between prolonged exposure to long-working hours and stroke subtypes in the constances cohort. Occup. Med..

[36] Liu Q., Wang X., Wang Y., Wang C., Zhao X., Liu L., Li Z., Meng X., Guo L., Wang Y. (2018). Association between marriage and outcomes in patients with acute ischemic stroke. J. Neurol..

[37] Gao C., Roberts J.A., Rundek T., Elkind M.S.V., Gardener H., Gutierrez J. (2024). Marital status and vascular events in a diverse northern manhattan cohort. Innov. Aging.

[38] Aron A.W., Staff I., Fortunato G., McCullough L.D. (2015). Prestroke living situation and depression contribute to initial stroke severity and stroke recovery. J. Stroke Cerebrovasc. Dis..

[39] Xia N., Li H. (2018). Loneliness, Social Isolation, and Cardiovascular Health. Antioxid. Redox Signal..

[40] Sawamura S., Enya A. (2023). A retrospective study on return to living alone of stroke patients who were living alone before stroke. J. Phys. Ther. Sci..

[41] Larsson S.C., Burgess S., Michaëlsson K. (2019). Smoking and stroke: A mendelian randomization study. Ann. Neurol..

[42] Falkstedt D., Wolff V., Allebeck P., Hemmingsson T., Danielsson A.-K. (2017). Cannabis, Tobacco, Alcohol Use, and the Risk of Early Stroke: A Population-Based Cohort Study of 45 000 Swedish Men. Stroke.

[43] Smyth A., O’Donnell M., Rangarajan S., Hankey G.J., Oveisgharan S., Canavan M., McDermott C., Xavier D., Zhang H., Damasceno A. (2023). Alcohol Intake as a Risk Factor for Acute Stroke: The INTERSTROKE Study. Neurology.

[44] Lin Q., Ye T., Ye P., Borghi C., Cro S., Damasceno A., Khan N., Nilsson P.M., Prabhakaran D., Ramirez A. (2022). Hypertension in stroke survivors and associations with national premature stroke mortality: Data for 2·5 million participants from multinational screening campaigns. Lancet. Glob. Health.

[45] Mezhal F., Oulhaj A., Abdulle A., AlJunaibi A., Alnaeemi A., Ahmad A., Leinberger-Jabari A., Al Dhaheri A.S., AlZaabi E., Al-Maskari F. (2023). High prevalence of cardiometabolic risk factors amongst young adults in the United Arab Emirates: The UAE Healthy Future Study. BMC Cardiovasc. Disord..

[46] Aljafen B.N., Meo A.S., Shaikh N., Meo S.A. (2025). Stroke incidence, mortality and disability-adjusted life years (DALYs) trends in association with air pollution, dietary and metabolic risk factors in Gulf Cooperation Council countries: Global burden of disease data based analysis 1990–2021. Saudi Med. J..

[47] Deedi M.K. (2017). Role of Anthropometric Measurements in Development of CVD and Stroke among T2DM in East Godavari District, Andhra Pradesh, India. J. Clin. Diagn. Res..

[48] Bukhari S., Yaghi S., Bashir Z. (2023). Stroke in Young Adults. JCM.

[49] Ellis C. (2010). Brief Report Stroke in young adults. Disabil. Health J..

[50] Liu Q., Su J., Liang Y., He X. (2025). Global burden and trend of stroke attributable to metabolic risks among young adults (20–39 years old) from 1990 to 2021. Front. Cardiovasc. Med..

[51] Zrelak P.A. (2024). Evolving Evidence Practices in Acute Stroke Care. Int. J. Crit. Care.

[52] Crane M.M., Halloway S., Walts Z.L., Gavin K.L., Moss A., Westrick J.C., Appelhans B.M. (2021). Behavioural interventions for CVD risk reduction for blue-collar workers: A systematic review. J. Epidemiol. Community Health.

[53] Sič A., Andrejić N., Ivanović J., Karadžić Ristanović V., Gajić S., Bjelić D., Baralić M., Stojanovic N. (2025). Stroke in Young Adults: An Overview and Non-Pharmacological Preventive Strategies. Brain Sci..

[54] Alshehri A. (2019). Stroke in atrial fibrillation: Review of risk stratification and preventive therapy. J. Fam. Community Med.

[55] AlAmeri M., AlNuaimi A., Alsaadi T. (2015). Stroke in Young Adults: A 4-Year Retrospective Hospital-Based Study, First Report From United Arab Emirates (P1.020). Neurology.

[56] Fekadu G., Adola B., Mosisa G., Shibiru T., Chelkeba L. (2020). Clinical characteristics and treatment outcomes among stroke patients hospitalized to Nekemte referral hospital, western Ethiopia. J. Clin. Neurosci. Off. J. Neurosurg. Soc. Australas..

